# Outcomes of patients with COVID-19 and coronary artery disease and heart failure: findings from the Philippine CORONA study

**DOI:** 10.1186/s13104-023-06677-5

**Published:** 2024-01-04

**Authors:** Adrian I. Espiritu, John Christopher A. Pilapil, Jaime Alfonso M. Aherrera, Marie Charmaine C. Sy, Veeda Michelle M. Anlacan, Emilio Q. III Villanueva, Roland Dominic G. Jamora

**Affiliations:** 1 Department of Neurosciences, College of Medicine and Philippine General Hospital, University of the Philippines Manila, Manila, Philippines; 2 Department of Clinical Epidemiology, College of Medicine, University of the Philippines Manila, Manila, Philippines; 3 Division of Cardiovascular Medicine, Department of Medicine, College of Medicine, Philippine General Hospital, University of the Philippines Manila, Manila, Philippines; 4 Department of Pathology, College of Medicine and Philippine General Hospital, University of the Philippines Manila, Manila, Philippines; 5 Institute for Neurosciences, St. Luke’s Medical Center, Global City, Philippines

**Keywords:** COVID-19, Coronary artery disease, Heart failure, Mortality, Respiratory failure, Intensive care unit admission, Hospital stay

## Abstract

**Background:**

Patients with coronavirus disease 2019 (COVID-19) and coronary artery disease (CAD) or heart failure (HF) are more likely to have poor outcomes. This study aimed to determine the characteristics and outcomes of COVID-19 patients with CAD/HF across various institutions in the Philippines.

**Methods:**

We utilized the data from the Philippine CORONA Study and compared the outcomes of admitted COVID-19 patients with CAD/HF versus those without. The Student’s t test, Mann-Whitney U test, binary logistic regression and multivariate regression analysis were utilized. Odds ratios (OR) and Kaplan-Meier curves were generated.

**Results:**

We included a total of 512 patients with COVID-19 had CAD/HF and 10,369 were without. CAD/HF was significantly associated with COVID severity, all-cause mortality, death from cardiac causes, respiratory failure, and prolonged hospitalization. After adjusting for confounders, the presence of CAD/HF was still associated with death from a cardiac cause (OR 2.22, 95% CI 1.49–3.3, *p* < 0.01).

**Conclusions:**

The presence of CAD or HF was significantly associated with severity of COVID disease, all-cause mortality, death from cardiac causes, respiratory failure, and prolonged hospitalization.

**Supplementary Information:**

The online version contains supplementary material available at 10.1186/s13104-023-06677-5.

## Introduction

Coronavirus disease 2019 (COVID-19) is caused by the SARS-CoV-2 virus. More severe presentations of COVID-19 may include pneumonia and acute respiratory distress syndrome (ARDS). As of September 30, 2023, over 695 million individuals have been affected by the virus with about 6.9 million deaths [1]. In the Philippines, a total of 3.6 million have been diagnosed since the start of the pandemic, with over 60,000 registered deaths from the infection [2].

The cardiovascular system and cardiovascular diseases (CVD) have been increasingly implicated in COVID-19, both as a risk for infection and for worse outcome of the disease [3]. There is substantial evidence of an association between CVD risk factors such as hypertension, diabetes, prior coronary artery disease (CAD) and the risk and severity of COVID [3, 4]. A previous meta-analysis showed that in comparing severe and non-severe patients, the pooled odds ratio (OR) of cardiovascular disease was 3.42 (95% confidence interval (CI) 1.88–6.22) and hypertension was 2.36 (95% CI: 1.46–3.83) [5]. Two underlying cardiovascular diseases, CAD and heart failure (HF), have been commonly implicated with worse outcomes of COVID-19 patients [3–6].

CAD prevalence possesses variations from population-to-population. Thus, among patients with COVID-19, there is a wide range for the prevalence of CAD, reported to be between 4.2 and 25% [3, 4, 7, 8]. Published data suggests that history of CAD in COVID-19 patients is associated with more severe disease and risk for death, but most studies to this date are retrospective in nature [9]. Data also suggests that among COVID patients requiring admission, more patients have a history of CAD and even among those who died, the percentage possessing CAD is likewise higher [8, 9].

Similar to CAD, HF has been identified as both a portal to and sequelae of severe COVID disease. In patients with COVID-19, HF may be precipitated by acute illness in those with pre-existing known or undiagnosed heart disease (e.g. hypertensive heart disease, CAD), acute hemodynamic stress (e.g. right ventricular failure from acute pulmonary hypertension and severe ARDS), or new onset myocardial injury (e.g. acute myocardial infarction (MI), stress or infection-related cardiomyopathy, cytokine storm) [10–12]. Knowledge on the incidence of COVID-19 patients with pre-existing HF has been less studied compared to those with pre-existing CAD [3, 8]. Nonetheless, similar to the general trends for CVD, a history of HF has likewise been associated with increased odds for mortality, risk for mechanical ventilation, and longer length of hospital stay [13].

COVID-19 remains to be a global problem, demanding a significant burden on healthcare systems and socioeconomic structures, notably on lower middle income countries like the Philippines. The Philippine CORONA study initially aimed to determine the effects of new onset neurological symptoms on clinically relevant outcomes in hospitalized patients with COVID-19 infection [14, 15]. This study aimed to look into the characteristics and outcomes of those patients with CAD or HF to further shed light and information on the effects of CVD on patients who contract COVID-19 and vice versa.

## Methods

### Study design

An analysis of patients diagnosed with CAD or HF was performed based on the data from the Philippine CORONA Study [14, 15]. The complete protocol of the Philippine CORONA study was approved and endorsed by the Single Joint Research Ethics Board of the Philippine Department of Health and the other respective institutions [14]. Data was obtained entirely from a retrospective review of medical records. The protocol was also registered in ClinicalTrials.gov (NCT04386083).

### Setting

A total of 37 major hospitals and study sites from various regions in the Philippines were included in the study. The complete list of study sites are listed below [15].

### Patient selection, sampling and cohort description

All patients analyzed in the Philippine CORONA Study were included in the analysis [15]. Adult COVID-19 patients with diagnosed CAD or HF were designated as the exposed cohort, while those without CAD or HF were grouped as part of the unexposed cohort. As stipulated in the protocol of the original CORONA study, the diagnoses of both CAD and HF were based on retrospective review of patients’ medical records [14].

### Outcome variables

The outcome variables of interest were similar to that of the Philippine CORONA Study: mortality, respiratory failure, duration of ventilator dependence, intensive care unit (ICU) admission, length of ICU stay, and length of hospital stay [15].

### Sample size

A total of 10,881 patients were included in the final analysis. Among these, 512 were diagnosed to have CAD or HF while 10,369 were without CAD or HF [15].

### Statistical analysis

Baseline characteristics and clinical outcomes of the participants were summarized by descriptive statistics. Numerical variables were described as mean and standard deviation (SD), if the data was normally distributed as assessed by Shapiro-Wilk test for normality, and as median and interquartile range (IQR), if otherwise. Categorical variables were described as count and proportion. The various baseline characteristics and clinical outcomes of interest were compared between the two groups: with the composite of CAD or HF and without CAD or HF. For normally distributed data, the Student’s t test was employed to determine significant differences in the mean, median, and mean-rank of the numerical variables between the two groups. The Mann-Whitney U test was utilized for non-normally distributed variables. The Chi-square test or Fisher exact test was used to determine heterogeneity of various categorical variable proportions between the two cohorts.

The associations between CAD/HF and the different individual dichotomous outcome variables of interest were determined by binary logistic regression. Survival analysis was performed for time-to-event data of mortality, mortality from cardiac cause, and respiratory failure. The time-to-event were right-censored on time-to-discharge as the exit from the time-at-risk among those who have not experienced the event, i.e., mortality, or respiratory failure, during the hospital stay. Multivariable Cox proportional hazards regression was utilized to determine the associations between having CAD/HF and the different time-to-event outcome variables of interest. Multivariable regression analysis was performed to adjust for age, sex, comorbidities, and treatment received with significant differences between the two groups. A cutoff of *p* value < 0.05 was set to identify CAD/HF as significant predictor of the different outcomes of interest. Kaplan-Meier curves were constructed to visualize the failure plot of the full cohort, and comparison between patients with versus without CAD/HF for death from cardiac cause.

## Results

### Inclusion of patients

Patients diagnosed with COVID-19 who likewise had underlying CAD and HF were identified from 10,881 patients were included in the final analyses. Among those, 512 were found to have either CAD or HF. The remaining 10,369 patients had neither CAD nor HF.

### Baseline characteristics of COVID-19 patients with CAD or HF

The median age of the CAD/HF cohort was 61 years, significantly older than the non-CAD/HF group which had a median age of 51 years. The CAD/HF population was predominantly male. Compared to the non-CAD/HF group, the CAD/HF cohort had statistically significant more comorbidities and risks, including history of smoking, hypertension, diabetes, kidney disease, and stroke. There was no statistically significant difference in the proportion of obese patients between the two groups. Severe or critical COVID-19 disease severity was more prevalent among those with CAD/HF (62.48%, n = 318 vs. 36.55%, n = 3743; *p* < 0.001), while mild-moderate disease was more prevalent among individuals who did not have CAD/HF (37.52%, n = 191 vs. 63.45%, n = 6499; *p* < 0.001). A higher proportion of the CAD/HF cohort received more aggressive management such as tocilizumab, systemic glucocorticoids, remdesivir, and antiplatelets. There was no difference in the proportion given anticoagulants and undergoing hemoperfusion between the two groups, though. Table 1 summarizes the clinicodemographic characteristics of our cohort, stratified by the presence or absence of CAD/HF.

**Table 1:** Study population baseline clinicodemographic characteristics stratified by coronary artery disease or heart failure diagnoses

	With CAD/HF	Without CAD/HF	*p* value
	n = 512	n = 10, 369	
**Sociodemographic data**			
Age (IQR)	64 (18.5)	51 (29)	< 0.001
Sex			< 0.001
*Male*	331 (64.65%)	5449 (52.56%)	
*Female*	181 (35.35%)	4918 (47.44%)	
Nationality			0.023
*Filipino*	512 (100.00%)	10,277 (99.11%)	
*Others*	0	92 (0.89%)	
Body mass index^a^	24.61 (5.26)	24.98 (6.08)	0.145
**Comorbid diseases and risks** **Non-neurologic**			
Smoking history	108 (21.09%)	918 (8.85%)	< 0.001
Hypertension	411 (80.27%)	3236 (31.21%)	< 0.001
Diabetes	261 (50.98%)	1930 (18.61%)	< 0.001
Chronic obstructive pulmonary disease	29 (5.66%)	127 (1.22%)	< 0.001
Bronchial asthma	20 (3.91%)	443 (4.27%)	0.689
Dyslipidemia			
Kidney disease	110 (21.48%)	501 (4.83%)	< 0.001
Obesity^a^	36 (14.81%)	729 (17.05%)	0.366
Healthcare worker	17 (3.32%)	859 (8.28%)	< 0.001
**Neurologic history/ Chronic neurologic disease**			
Stroke	57 (11.13%)	264 (2.55%)	< 0.001
Epilepsy	0	27 (0.26%)	0.637
Others	10 (1.95%)	62 (0.60%)	< 0.001
**COVID severity**			< 0.001
Severe/Critical	318 (62.48%)	3743 (36.55%)	
Mild/Moderate	191 (37.52%)	6499 (63.45%)	
**Treatments received**			
Tocilizumab	83 (16.21%)	946 (9.12%)	< 0.001
Steroids	242 (47.27%)	2602 (25.09%)	< 0.001
Remdesivir	128 (25.00%)	1216 (11.73%)	< 0.001
Anticoagulation	54 (10.55%)	977 (9.42%)	0.396
Antiplatelets	15 (2.93%)	48 (0.46%)	< 0.001
Convalescent plasma therapy	24 (4.69%)	239 (2.30%)	0.001
Hemoperfusion	10 (1.95%)	115 (1.11%)	0.080

^a^Only 4518 participants have body mass index data and corresponding obesity information

CAD – coronary artery disease; HF – heart failure; IQR – interquartile range

### Effect of CAD/HF on outcomes of COVID-19 patients in the Philippine CORONA Study

In terms of clinically relevant outcomes, most deaths from any cause occurred in the CAD/HF group. Among the non-cardiac causes of mortality, only multiorgan dysfunction was more prevalent in the CAD/HF group; while the rest occurred with the same frequency between the two groups. Death from a cardiac cause was also more frequent among those with CAD/HF. In terms of neurological outcomes, full/partial improvement was more frequent in those without CAD/HF. Respiratory failure was also more frequent in the CAD/HF group. Table 2 summarizes the clinical outcomes of our cohort, stratified by the presence/absence of CAD/HF.

**Table 2:** Comparison of clinical outcomes in COVID-19 patients included in the Philippine CORONA Study stratified by coronary artery disease or heart failure diagnoses

	With CAD/HF	Without CAD/HF	*p* value
	(n = 512)	(n = 10,369)	
**All-cause mortality**			< 0.001
Mortality, n (%)	153 (29.88%)	1549 (14.94%)	
Survivor, n (%)	359 (70.12%)	8820 (85.06%)	
**Death from cardiac cause** ^**a**^	45 (29.41%)	213 (13.75%)	< 0.001
**Non-cardiac causes of mortality**			
Acute respiratory distress syndrome, n (%)	58 (37.91%)	662 (42.74%)	0.249
Septic shock, n (%)	55 (35.95%)	632 (40.80%)	0.243
Brain herniation, n (%)	7 (4.58%)	59 (3.81%)	0.640
Multiorgan dysfunction, n (%)s	23 (15.03%)	120 (7.75%)	0.002
Others, n (%)	16 (3.12%)	180 (1.74%)	0.021
**Neurologic outcomes** ^**b**^			< 0.001
Full/partial improvement, n (%)	96 (74.42%)	1543 (86.88%)	
Stable, no improvement, n (%)	33 (25.58%)	233 (13.12%)	
**Respiratory failure, n (%)**	169 (75.7%)	1439 (13.88%)	< 0.001
**Admitted to ICU, n (%)** Length of ICU stay in days^c^, median (IQR)	15 (11)	15 (12)	0.670
**Length of hospital stay in days, median (IQR)**	14 (11)	13 (9)	< 0.001

^a^Cardiac causes of death are acute coronary syndrome, decompensated heart failure, and cardiac arrhythmia

^b^Only 1905 participants have neurologic outcome data among the 2291 participants with neurologic presentations and/or complications

^c^Among 1740 participants who have been admitted in the ICU

CAD – coronary artery disease; HF – heart failure; ICU – intensive care unit

### Estimation of CAD/HF association with clinically relevant outcomes

**Fig. 1:**
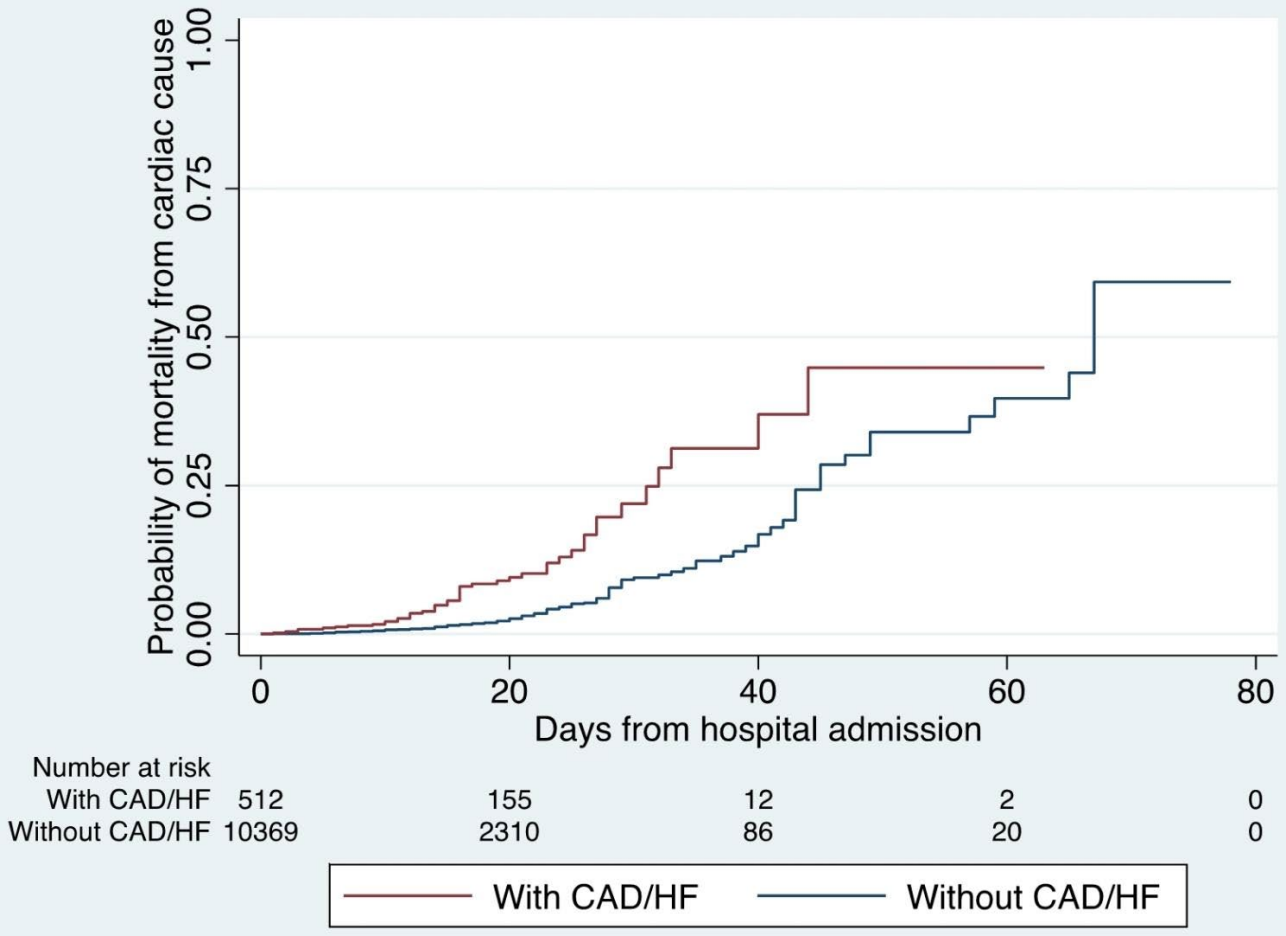
Kaplan-Meier failure plot of death from cardiac cause of the full Philippine CORONA, comparing between COVID-19 patients with and without CAD/HF

The presence of CAD/HF was significantly associated with COVID severity (OR 2.89, 95% CI 2.41–347, *p* < 0.001), all-cause mortality (OR 2.43, 95% CI 1.99–2.95, *p* < 0.001), death from cardiac causes (OR 2.61, 95% CI 1.79–3.81, *p* < 0.001), respiratory failure (OR 3.06, 95% CI 2.52–3.71, *p* < 0.001), and prolonged hospitalization (OR 1.43, 95% CI 1.19–1.7, *p* < 0.001). After adjusting these with other confounders on multivariate analysis (age, sex, smoking history, comorbidities, and therapeutics received), the presence of CAD/HF was still associated with death from a cardiac cause (OR 2.22, 95% CI 1.49–3.3, *p* < 0.01). Table 3 summarizes the association of CAD/HF with clinically relevant outcomes. On Kaplan-Meier plot analysis (see Fig. 1), those with CAD/HF had a poorer survival (i.e., dying from any cardiac cause) compared to those without CAD/HF. Detailed results pertaining to each clinically relevant outcome may be found in Supplementary material 1 **Appendix 1**.

**Table 3:** Association of history coronary artery disease or heart failure to various outcomes of interest among COVID-19 patients in the Philippine CORONA Study

Outcomes	Estimate^a^	95% CI	*p* value	Adjusted Estimate^b^	95% CI	*p* value
**Dichotomous outcomes**						
Severe/critical COVID-19^c^	2.89	2.41, 3.47	< 0.001			
Full/partial improvement of neurologic deficit/s	0.44	0.29, 0.67	< 0.001	0.94	0.59, 1.51	0.799
All-cause mortality	2.43	1.99, 2.95	< 0.001	1.06	0.85, 1.33	0.577
Death from cardiac cause	2.61	1.79, 3.81	< 0.001	2.22	1.49, 3.32	< 0.001
Respiratory failure	3.06	2.52, 3.71	< 0.001	1.21	0.97, 1.51	0.084
Prolonged duration of ventilator dependence	0.48	0.30, 0.77	0.002	0.99	0.60 1.62	0.957
Prolonged length of ICU stay	1.39	0.89, 2.17	0.150	1.58	1.00, 2.52	0.052
Prolonged length of hospital stay	1.43	1.19, 1.70	< 0.001	1.16	0.96, 1.40	0.124
**Time-to-event outcomes**						
All-cause mortality	1.43	1.21, 1.69	< 0.001	0.98	0.83, 1.17	0.846
Death from cardiac cause	3.02	2.18, 4.18	< 0.001	1.91	1.34, 2.71	< 0.001
Respiratory failure	2.59	2.20, 3.03	< 0.001	1.07	0.90, 1.26	0.437

^a^Odds ratio for dichotomous outcomes, and hazard ratio for time-to-event outcomes

^b^Adjusted for age, sex, smoking history, comorbidities with hypertension, diabetes mellitus, chronic obstructive pulmonary disease, bronchial asthma, kidney disease, and stroke, and, receiving tocilizumab, steroid, anti-platelet, remdesivir, and covalescent plasma therapy.

COVID – Coronavirus disease; ICU – intensive care unit; CI – confidence interval

## Discussion

The Philippine CORONA study was the largest in the country, involving 10,881 hospitalized COVID-19 patients [15]. This study looked into the association of CAD/HF history with baseline characteristics and clinical outcomes among COVID-19 patients.

Patients diagnosed with CAD or HF who were hospitalized for COVID-19 composed 4.7% of our entire study population. This was consistent with published data that shows for CAD, the prevalence among COVID-19 patients ranges from around 4–25% [6, 7, 9]. Data for pre-existing HF among COVID-19 patients has been lacking, as most studies have been instead designed to report the incidence of new onset HF in hospitalized patients [3, 4, 7, 8]. Our CAD/HF cohort was significantly older with a median of 61 years, 10 years more than the non-CAD/HF group and was predominantly male. These are consistent with the general trends for patients with CVD [16]. It is important to note, however, that older age and male sex have been postulated as individual risk factors as well for severe COVID and worse clinical outcomes [16, 17]. In our study, patients with CAD/HF had more prevalent comorbidities and clinical risk factors such as smoking history, hypertension, diabetes, kidney disease, and stroke. This appears to be logical, as all of the aforementioned factors have been consistently shown to be well correlated with CVD, inclusive of CAD and HF. None of our included patients had received prior vaccination for COVID-19. This was because at the time data was collected for the Philippine CORONA study, vaccination for COVID-19 was not yet available in the Philippines.

A number of studies have likewise shown that the above risk factors are independently associated with adverse outcomes in COVID-19 such as increased disease severity, all-cause mortality, respiratory failure, and longer length of hospitalization and/or need for ICU admission [7, 8, 18–21]. Previously published subgroup analyses that looked into diabetic patients of the Philippine CORONA study reported similar data and trends [22]. Consistent findings were likewise noted in patients with hypertension and chronic obstructive pulmonary disease in the Philippine CORONA study, with hypertension and chronic obstructive pulmonary disease being found to have increased odds for in-hospital mortality, respiratory failure, ICU admission, and severe/critical COVID-19 disease [23, 24]. Meanwhile, COVID-19 stroke patients in the Philippine CORONA population had higher mortality and ICU admission rates compared to those with COVID-19 alone or those with COVID-19 and stroke from developed countries [25]. In the same population, it was reported that being underweight was a predictor of prolonged invasive mechanical ventilation requirement and that obesity (using the Asia-Pacific cutoffs) correlated with the need for ICU admission [26]. Any body mass index abnormality actually correlated with increased odds of severe/critical COVID-19 [26]. Finally, it is worthy to note that in the Philippine CORONA study, cancer – which has long been postulated as a non-cardiovascular risk factor – likewise conferred worse clinical outcomes and more severe disease in COVID-19 afflicted patients [27].

Although more well known as a disease of the respiratory tract, COVID-19 has also been postulated to directly and indirectly affect the cardiovascular system (MI, HF, cardiac arrhythmias, sudden cardiac death, venous thromboembolism such as deep venous thrombosis and pulmonary embolism, and arterial embolism) [4–8, 18, 19]. The mechanisms of injury to the cardiovascular system are inclusive of direct myocardial injury from hemodynamic derangement and/or hypoxemia, inflammatory myocarditis, stress cardiomyopathy, microvascular dysfunction or thrombosis due to hypercoagulability, or systemic inflammation and cytokine storm [4, 28]. A number of these pathways may destabilize coronary artery plaques.

Studies have previously suggested that COVID-19 increased the risk for acute MI [29–31]. In an analysis of 3334 patients hospitalized for COVID-19 in New York, it was shown that compared with non-infected controls, the risk for acute MI was increased in patients newly diagnosed with COVID-19. Furthermore, all-cause mortality was around 22% higher in those who suffered from thrombotic events. In their study, after multivariable adjustment, any thrombotic event was independently associated with mortality, regardless of whether the thrombosis was venous or arterial [31, 32]. Another study showed that myocardial injury is significantly associated with fatal outcomes, cardiac dysfunction, and arrhythmias for COVID-19 patients, but the prognosis of patients with underlying CVD who did not develop myocardial injury was relatively favorable [32, 33].

In our study, the presence of CAD/HF was significantly associated with severe or critical COVID disease severity and consequently, increased all-cause mortality. A pooled analysis on COVID-19 and CAD reveals that the odds of CAD occurrence was lower among COVID-19 survivors vs. non-survivors and the presence of CAD was significantly associated with severe disease [9]. In an analysis of 6439 patients hospitalized with COVID-19 in New York hospitals, it was determined that a history of HF was associated with increased mortality. Interestingly, outcomes across patients with different types of HF were similar, regardless of left ventricular ejection fraction [13].

Our data on patients of the Philippine CORONA Study likewise showed that patients with CAD/HF were at increased risk for death from cardiac causes, including acute coronary syndrome, decompensated HF, and fatal cardiac arrhythmias. While there is already supporting evidence that COVID-19 may indeed increase the risk for cardiovascular death and events, specific data regarding death from cardiac causes for patients with pre-existing CAD or HF – such as the increased correlation reported by this study – is still wanting [3–9, 18, 19]. It does stand to reason, however, that because of the deleterious effects it exerts on the cardiovascular system, COVID-19 may to increased cardiovascular events and subsequently, cardiovascular death. The results of our study affirm this as even after adjusting for other confounders (age, sex, smoking history, comorbidities, and therapeutics received), the presence of CAD/HF was still associated with increased death from a cardiac cause (OR 2.22, 95% CI 1.49–3.3, *p* < 0.01).

Finally, results of our study show that the presence of CAD/HF was associated with increased risk for respiratory failure and longer length of hospitalization. This was consistent with prior data showing that those with previous HF have increased risk for mechanical ventilation and experienced longer length of stay. Apart from being independent from baseline ejection fraction, the aforementioned outcomes were similar regardless of renin-angiotensin-aldosterone inhibitor use [13].

The association of a prior history of CAD/HF with increased all-cause mortality, cardiovascular death, respiratory failure, and length of hospitalization for our study’s patients with COVID-19 underscores the burden of CVD on clinical outcomes. The fact that, even adjusting for other comorbidities and treatments administered, death from a cardiac cause is still increased among CAD/HF patients is a testament that the combination of COVID-19 and CVD in a patient makes for less favorable results. It also highlights the increasing need for our healthcare system to address primordial and primary mitigating strategies that will reduce the incidence of both CAD and HF in our already vulnerable population. For those already with CAD/HF, our study showed that it is paramount to employ strategies that prevent them from being infected with COVID-19, such as vaccination and booster prioritization and added vigilance in preventing adverse clinical outcomes should they be admitted for COVID-19 infection.

### Limitations

This study has several limitations. The diagnosis of both CAD and HF were both obtained via medical records review. No formal criteria involving symptomatology, physical examination findings, non-invasive imaging, and invasive testing was employed in determining the presence of both diseases, both of which were entrusted to the diagnoses of attending physicians on retrospective review of patient charts. This study likewise did not specify whether CAD/HF patients had previous acute coronary syndrome, revascularization, decompensation and/or hospitalization for heart failure; nor did it detail the subtypes of CAD/HF. Data for the occurrence of venous thromboembolism such as deep venous thrombosis and pulmonary embolism was not recorded. For future studies, it would likewise be useful to determine and obtain data on pertinent parameters for coronary artery disease and heart failure such as functional class (e.g. Canadian Cardiovascular Society class for angina and New York Heart Association class for HF), ischemia burden, involved epicardial coronary arteries on angiogram, ejection fraction and other echocardiographic parameters, baseline and in-hospital cardiac troponins and NT pro-BNP levels, if available. Finally, the included population was only that of hospitalized COVID-19 patients. The absence of data from COVID-19 patients who were managed at the outpatient level possibly overestimates adverse clinical outcomes such as all-cause mortality, cardiac death, and respiratory failure, as those with severe or critical disease are more likely to have been hospitalized.

## Conclusion

Among COVID-19 patients, the presence of CAD or HF was significantly associated with severity of COVID disease, all-cause mortality, death from cardiac causes, respiratory failure, and prolonged hospitalization. Its association with death from a cardiac cause was still significant even after adjusting for other confounders.

### Electronic supplementary material

Below is the link to the electronic supplementary material.

Supplementary Material 1

## Data Availability

The datasets used and/ or analyzed during the current study are available from the corresponding author on reasonable request.

## Declarations

This study was performed in line with the principles of the Declaration of Helsinki and adhered to the Philippine National Ethical Guidelines for Health and Health-related Research 2017. Acquiring informed consent was waived by all the institutional review boards since the design was a retrospective cohort study employing medical chart review and the data obtained for this study were completely anonymized.

## Abbreviations

ARDS: Acute respiratory distress syndrome
CAD: Coronary artery disease
CI: Confidence interval
COVID-19: Coronavirus disease 2019
CVD: Cardiovascular diseases
HF: Heart failure
ICU: Intensive care unit
IQR: Interquartile range
MI: Myocardial infarction
OR: Odd’s ratio
SD: Standard deviation
