# Supplementary material for: Outcomes of patients with COVID-19 and coronary artery disease and heart failure: findings from the Philippine CORONA study
Source: BMC Res Notes. 2024 Jan 4;17:14. doi: 10.1186/s13104-023-06677-5 (PMC10768280; doi:10.1186/s13104-023-06677-5)
Supplement: Supplementary file 1 — Supplementary Material 1 [file 13104_2023_6677_MOESM1_ESM.docx]

**APPENDIX**

*All-cause mortality.* More deaths from any cause occurred in the CAD/HF cohort (29.88%, n = 153) compared to the non-CAD/HF group (14.94%, n = 1549; *p* < 0.001). The presence of CAD/HF was found to be associated with increased mortality from any cause (OR 2.43, CI 1.99-2.95, *p* < 0.001). When adjusting for other confounders (age, sex, comorbidities, therapeutics received), however, there was no statistically significant difference in terms of all-cause mortality between the two cohorts (OR 1.08, 95% CI 0.86-1.34, *p* = 0.514). Subsequent time-to-event analysis, shows that those with CAD/HF had a poorer survival (i.e., dying from any cardiac cause) compared to those without CAD/HF.

*Cardiac death.* The CAD/HF cohort had a higher prevalence of death from cardiac cause (e.g. acute coronary syndrome, decompensated heart failure, cardiac arrhythmia) compared with the group without CAD/HF (29.41%, n = 45 vs. 13.75%, n = 213; *p* < 0.001). CAD/HF was thereby associated with higher odds of dying of cardiac causes (OR 2.61, 95% CI 1.79-3.81, *p* < 0.001). A similar trend was noted even when adjusting for confounders (OR 2.22, 95% CI 1.49-3.30; *p* < 0.001).

*Neurologic utcomes.* Significantly more patients with CAD/HF had no improvement in neurologic outcomes compared to those without CAD/HF (25.28%, n = 33 vs. 13.12%, n = 233; *p* < 0.001). Likewise, among patients with neurologic dysfunction, a higher proportion of patients with no CAD/HF (86.88%, n = 1543) tended to have either full or partial improvement compared to patients with CAD/HF (74.42%, n = 96; *p* <0.001). Overall analysis shows that CAD/HF leads to decreased odds of neurologic recovery or improvement (OR 0.44, 95% CI 0.29-0.67; *p* < 0.001). After adjusting for confounders, however, no difference was seen between the two groups (OR 0.91, 95% CI 0.57-1.46; *p* = 0.703).

*Respiratory failure.* More patients with CAD/HF had respiratory failure (75.7%, n = 169) compared to patients in the non-CAD/HF group (13.88%, n = 1439; *p* < 0.001). OR for respiratory failure was higher in the CAD/HF group (OR 3.06, 95% CI 2.52-3.71, *p* < 0.001), but the noted effect was not preserved after adjusting for confounders (OR 1.23, 95% CI 0.98-1.53).

*Length of ICU stay.* Among patients admitted to the ICU, there was no significant difference in median length of ICU stay between the CAD/HF and non-CAD/HF cohorts (median 15, IQR 11 vs. median 15, IQR 12, *p* = 0.670). There was insufficient evidence to determine whether CAD/HF leads to increased odds for prolonged length of ICU stay (OR 1.39, 95% CI 0.89-2.17; *p* = 0.150).

*Length of hospital stay.* Median length of hospital stay was longer in patients with CAD/HF compared to those without (median 14, IQR 11 vs. median 13, IQR 9; *p* < 0.001). The odds of prolonged length of hospital stay was higher in the CAD/HF group (OR 1.43, 95% CI 1.19-1.70; *p* < 0.001), but this difference was no longer observed after adjusting for confounders (OR 1.16, 95% 0.97-1.40; *p* = 0.113).
